# Self-Reported Eating Speed Is Dose-Dependently and Bidirectionally Associated with Long-Term Underweight- and Obesity-Related Weight-History Groups

**DOI:** 10.3390/nu18152412

**Published:** 2026-07-24

**Authors:** Katsumi Iizuka

**Affiliations:** Department of Clinical Nutrition, School of Medicine, Fujita Health University, Toyoake 470-1192, Japan; katsumi.iizuka@fujita-hu.ac.jp; Tel.: +81-562-93-2329

**Keywords:** underweight, obesity, self-reported eating speed, irregular meal timing, breakfast skipping

## Abstract

**Background:** Body mass index (BMI) changes across the life course, yet most studies rely on single time-point measures and focus primarily on obesity. Whether self-reported eating speed is associated with long-term weight-history groups, including persistent underweight, remains unclear. **Methods:** This retrospective cohort study used Japanese health checkup data from 564,198 participants. According to BMI history in their twenties, participants were classified into normal-weight, underweight-history, and obesity-history groups. The underweight-history and obesity-history groups were further stratified according to the proportion of BMI measurements obtained during the entire observation period that indicated the respective weight state (0–25%, 25–50%, 50–75%, and 75–100%). Repeated BMI measurements were analyzed using linear mixed-effects models to characterize longitudinal differences across weight-history groups. Associations of self-reported eating speed with these groups were examined using sex-stratified multivariable logistic regression models. **Results:** BMI differed clearly across weight-history groups in both sexes. Self-reported eating speed showed a dose-dependent and bidirectional association. In women, the odds of fast eating increased progressively with increasing obesity proportion, reaching an adjusted odds ratio (OR) of 1.47 (95% CI 1.40–1.53) in the 75–100% obesity group; in men, the corresponding OR was 2.40 (95% CI 2.35–2.45). Conversely, the odds of slow eating increased with increasing underweight proportion, reaching ORs of 1.89 (95% CI 1.82–1.96) in women and 2.45 (95% CI 2.35–2.56) in men in the 75–100% underweight group. In additional analyses, self-reported eating speed also showed persistence over follow-up: the mean proportion of follow-up responses matching the baseline self-reported eating-speed category was 72.33% (95% CI 71.25–73.42) in women and 79.90% (95% CI 79.55–80.25) in men in the 75–100% obesity group for fast eating, and 72.84% (95% CI 72.00–73.68) in women and 68.24% (95% CI 67.08–69.39) in men in the 75–100% underweight group for slow eating. **Conclusions:** Self-reported eating speed showed a consistent bidirectional association with long-term weight-history groups in both sexes and remained relatively persistent over follow-up. These findings suggest that self-reported eating speed may be a behavioral correlate of long-term weight-history groups.

## 1. Introduction

Obesity and underweight are multifactorial conditions with distinct but sometimes overlapping causes. Obesity is commonly linked to excess energy intake, low physical activity, genetic susceptibility, sleep disturbance, and adverse food environments [1]. Underweight may arise from insufficient energy intake, chronic disease, psychological factors, socioeconomic disadvantage, or constitutionally low body mass [2]. Both conditions can also be influenced by eating behaviors, lifestyle patterns, and developmental factors across the life course. Among these factors, eating behaviors have also been linked to body size. Previous studies have shown that late-evening meals and breakfast skipping are associated with obesity [3,4,5,6], and self-reported eating speed has likewise been linked to greater body size [7,8,9,10,11,12,13,14,15,16]. However, it remains unclear how these eating habits are related to long-term weight-history groups rather than BMI measured at a single time point alone. Thus, understanding these different pathways is important when examining long-term weight-history groups and their behavioral correlates.

Body mass index (BMI) is known to have a U-shaped association with health outcomes [17,18,19], indicating that both high and low body weight can be detrimental. Obesity has been associated with increased mortality and a wide range of morbidities, including cardiovascular disease, metabolic disorders, and malignancy [20,21,22]. Moreover, being underweight has attracted increasing attention, particularly in Japan, where the prevalence of underweight among young women is relatively high [3,23,24,25,26,27,28,29,30,31,32,33,34]. Previous studies have shown that underweight in young women is associated with reproductive and perinatal risks, including infertility and low birth weight [23,29]. In addition, I previously reported that young underweight individuals exhibit nutritional deficiencies, lower grip strength, reduced lymphocyte counts, and changes in the gut microbiota [30,31,32,33].

The proportions of obesity and underweight in Japan differ from those reported in many other countries. The prevalence of obesity among people in their twenties is 21.0% in men and 7.4% in women, and it increases with age, reaching 38.3% in men and 21.2% in women in their fifties [34]. Conversely, the prevalence of underweight in the twenties is 11.5% in men and 17.1% in women, but it decreases with age, falling to approximately 2.0% in men and 13.5% in women in their fifties [34]. Consistent with these observations, body weight is not static [33]. Even when a person is classified as underweight at a given time point, they may subsequently transition between underweight and normal weight. In our previous study of women in their twenties, I showed that those with underweight often experienced repeated transitions between weight states [33]. For example, approximately 60% later transitioned to normal weight, whereas about 40% remained underweight [33]. Thus, a diagnosis of underweight or obesity at a single time point may not adequately reflect the cumulative burden of that state [33]. Instead, the duration or accumulation of underweight and obesity may be more relevant to later health risks. In fact, recent studies have shown that cumulative exposure to underweight is associated with a higher risk of hip fracture [24], whereas a longer duration of obesity is associated with worsening cardiometabolic risk factors, including blood pressure, high-density lipoprotein cholesterol, and hemoglobin A1c [35,36,37,38]. These observations suggest that the persistence of abnormal weight states may have clinical significance beyond a single cross-sectional measure of body size alone, yet large-scale evidence from Japan remains limited.

Because BMI changes over time, a single measurement may not adequately capture long-term nutritional status or the cumulative burden of underweight and obesity [35,36,37,38]. Linear mixed-effects models have been widely used in trajectory research to evaluate longitudinal changes in body size; however, many previous studies have mainly focused on continuous increases or decreases in BMI and have paid less attention to clinically meaningful BMI thresholds [39,40]. Consequently, the persistence of underweight and obesity across follow-up may be overlooked when weight change is treated only as a continuous process. In the present study, I therefore classified participants according to the proportion of the observation period during which they remained underweight or obese, with the aim of capturing long-term weight-history groups in a way that reflects both longitudinal change and clinically relevant BMI categories.

Although I recognize the limitations of self-reported eating speed, including the possibility of subjective perception and misclassification, it remains a practical measure for large-scale epidemiological research. Among eating behaviors, eating speed has attracted particular interest because it is simple to assess and may reflect multiple aspects of eating behavior, including chewing duration, meal pace, and satiety-related responses [7,8,9,10,11,12,13,14,15,16]. Moreover, eating speed is often a habitual and largely unconscious aspect of eating behavior that may persist over time. Therefore, self-reported eating speed at the first health check-up in the twenties may still be informative as an early behavioral marker in relation to subsequent long-term weight-history groups. Previous studies have shown that faster self-reported eating speed is associated with higher BMI, obesity, and metabolic risk [7,8,9,10,11,12,13,14,15,16,41]. To complement the limitations of self-reported measures, I have previously measured eating speed directly and demonstrated its associations with chewing frequency and chewing tempo [42,43,44]. However, such direct measurements are feasible mainly in small-scale studies and are not practical for large population-based cohorts. Therefore, I was interested in whether eating speed at the first health check-up in the twenties was associated with subsequent long-term weight-history groups.

Thus, the aim of this study was to examine, separately in women and men, whether self-reported eating speed in the twenties was associated with subsequent long-term underweight- and obesity-related weight-history groups. I therefore compared subsequent body-size trajectories according to weight history in the twenties and examined how baseline self-reported eating speed differed across these long-term weight-history groups. By doing so, this study provides a framework for understanding long-term body weight regulation beyond BMI measured at a single time point and for examining how self-reported eating speed is related to persistent underweight- and obesity-related weight-history patterns.

## 2. Materials and Methods

### 2.1. Study Population and Definition of Weight-History Groups

**Figure 1:**
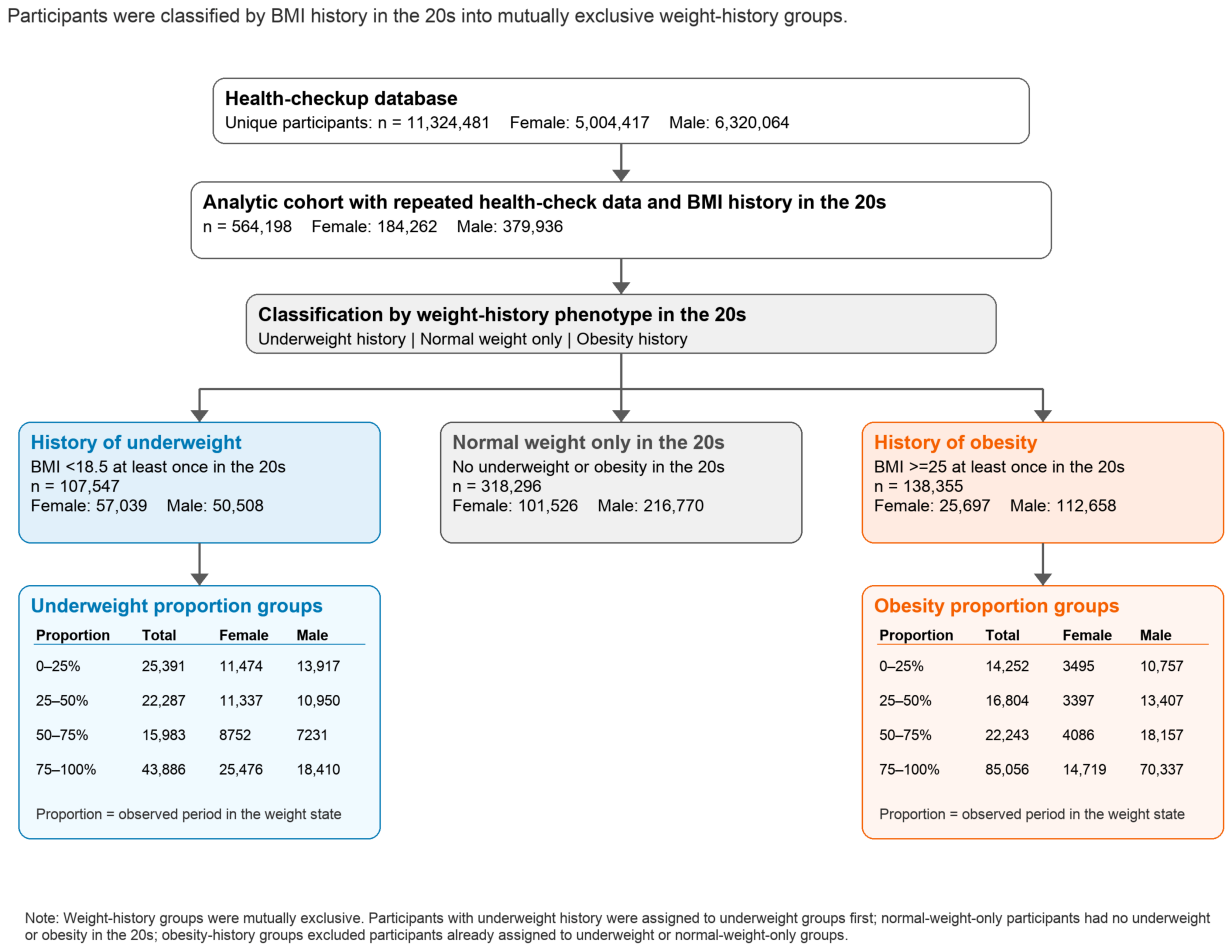
Flow diagram of retrospective cohort construction from the health check-up database.

This retrospective longitudinal cohort study used health check-up data purchased from JMDC, Inc. (Tokyo, Japan), covering Japanese health insurance beneficiaries between 2005 and 2025. We identified 11,324,481 individuals registered during this period, including 5,004,417 women and 6,320,064 men. Among them, 564,198 participants with at least five health check-up records were included in the analytic cohort (184,262 women and 379,936 men) (Figure 1). The JMDC health check-up database recorded participants as male or female, and no information on gender identity was available; therefore, sex rather than gender was assessed in the present study. Health check-up records in the JMDC database were generally available at approximately annual intervals, although the exact number and timing of records varied across participants.

According to BMI history during their twenties (defined as ages 20–29 years), participants were classified into those who had experienced underweight at least once (BMI < 18.5 kg/m^2^; history of underweight), those who had experienced obesity at least once (BMI ≥ 25.0 kg/m^2^; history of obesity), and those whose BMI remained within the normal-weight range throughout all health check-ups in that age period (normal weight only during the twenties). The underweight-history and obesity-history groups were further categorized according to the proportion of health check-up records across the entire observation period at which participants were classified as underweight or obese, respectively: 0–25%, 25–50%, 50–75%, and 75–100%. These proportion-based categories were selected to preserve clinically meaningful BMI thresholds while quantifying the persistence of underweight or obesity over the observed period. This proportion-based approach was adopted to reduce the influence of between-participant differences in the number of available health check-up records.

The observation period was defined individually for each participant as the interval from the first to the last available health check-up record in the database. Because participants entered the database in different calendar years, the cohort functioned as an open retrospective cohort, and follow-up duration therefore varied across individuals. Age was used as the analytical time scale in the trajectory models; therefore, participants were not required to share the same starting age or uniform follow-up intervals.

Participant counts are shown overall and by sex at each stage of cohort construction. Among 11,324,481 individuals registered in the health insurance database between 2005 and 2025, 564,198 participants with at least five health check-up records were included in the analytic cohort. Participants were classified according to BMI history during their twenties into three major groups: history of underweight, normal weight only during the twenties, and history of obesity. The underweight-history and obesity-history groups were further subdivided according to the corresponding underweight or obesity proportion: 0–25%, 25–50%, 50–75%, and 75–100%.

### 2.2. Questionnaire Items

The health checkup questionnaire included standardized items on lifestyle behaviors, including meal timing, sleep, exercise habits, physical activity, and walking speed. These variables were defined as follows: sleep adequacy was based on the self-reported response to whether the participant felt they obtained sufficient rest from sleep; habitual exercise was defined as exercise causing light sweating for at least 30 min per session at least 2 days per week for at least 1 year; physical activity was defined as walking or equivalent physical activity for at least 1 h per day; and walking speed was defined as self-reported walking speed faster than that of individuals of the same sex and similar age. These variables were coded as binary categorical variables.

### 2.3. Assessment of Eating Behaviors

Eating behaviors were assessed using a standardized questionnaire administered at routine health check-ups and were not developed specifically for the present study. Eating speed was based on self-reported perceptions of eating quickly, normally, or slowly. For self-reported eating-speed analyses, normal eating speed was used as the reference category. Late-evening meals were defined as eating dinner within 2 h before bedtime at least three times per week, and breakfast skipping was defined as skipping breakfast at least three times per week. Irregular meal timing was defined as the coexistence of late-evening meals and breakfast skipping. In the present study, self-reported eating speed, breakfast skipping, and late-evening meals were defined from the responses recorded at the first health check-up between the ages of 20 and 29 years and were treated as baseline behavioral variables. Thus, these questionnaire-based variables were measured in early adulthood and analyzed in relation to subsequent long-term weight-history groups and BMI trajectories.

### 2.4. Cross-Sectional BMI Categories at Age 35 Years

To illustrate the extent to which a single follow-up BMI category captured long-term weight-history groups, cross-sectional BMI categories at age 35 years were also examined according to the predefined weight-history groups. Among participants with an available BMI measurement at age 35 years, BMI was classified as underweight (<18.5 kg/m^2^), normal weight (18.5–24.9 kg/m^2^), or obesity (≥25.0 kg/m^2^). These categories were summarized separately for women and men across both the three major weight-history groups and the nine weight-history groups. When multiple BMI records were available at age 35 years for the same participant, the mean BMI at that age was used for classification.

### 2.5. Trajectory Analysis

BMI trajectories were evaluated using linear mixed-effects models, with age as the underlying time scale. Therefore, participants did not need to have measurements at the same starting age or at uniform follow-up intervals. Age was modeled using natural cubic spline terms. For the underweight-history and obesity-history groups, the models included fixed effects for weight-history group, spline terms for age, and their interaction, and were specified as follows:

BMIij = β0 + β1Groupi + f(Ageij) + Groupi × f(Ageij) + b0i + b1iAgeij + εij

where BMIij represents the BMI of participant i at visit j, Groupi represents the weight-history group, f(Ageij) denotes the natural cubic spline terms for age, b0i is the participant-specific random intercept, b1i is the participant-specific random slope for age, and εij is the residual error term. For the normal-weight-only group, the model included spline terms for age without group interaction. Models included participant-specific random intercepts and random slopes for age, specified as (1 + age || participant ID), allowing both baseline BMI and age-related change to vary across individuals. Estimated marginal means with 95% confidence intervals were then calculated at ages 25, 35, and 45 years. To formally assess whether trajectory patterns differed across groups over time, I additionally performed likelihood ratio tests comparing models with and without the interaction term between the weight-history group and age spline terms.

### 2.6. Association Between Self-Reported Eating Speed and Weight-History Groups

Associations between self-reported eating speed and long-term weight-history groups were examined using sex-stratified logistic regression models. Self-reported eating speed was defined from the first health check-up between ages 20 and 29 years and categorized as fast, normal, or slow. Separate models were fitted for fast eating and slow eating as the dependent variables, with weight-history groups entered as the main explanatory variables and the normal-weight-only group in the twenties used as the reference category. Models were adjusted for age and questionnaire-based covariates, including sleep adequacy, habitual exercise, physical activity, and walking speed. These variables were coded as binary categorical variables (1 = yes, 2 = no). As a sensitivity analysis, the models were further adjusted for irregular meal timing to assess whether the associations between self-reported eating speed and weight-history groups were independent of meal-timing behaviors. Adjusted odds ratios with 95% confidence intervals were calculated for each weight-history group.

### 2.7. Persistence of Self-Reported Eating Speed Across Weight-History Groups

As a supplementary analysis, I examined the persistence of self-reported eating speed during follow-up according to weight-history group. Participants were first classified according to their self-reported eating speed at the first health check-up between the ages of 20 and 29 years. For each participant, I identified all subsequent health check-ups with non-missing self-reported eating-speed responses and calculated the proportion of follow-up responses that matched the baseline self-reported eating-speed category. These participant-level matching proportions were then summarized according to weight-history group separately for women and men as mean percentages with 95% confidence intervals. For the fast-eating analysis, participants with baseline fast eating were compared across the normal-weight-only and obesity-related weight-history groups. For the slow-eating analysis, participants with baseline slow eating were compared across the normal-weight-only and underweight-related weight-history groups.

All analyses were conducted separately for women and men. All statistical analyses were performed in R version 4.5.3 (R Foundation for Statistical Computing, Vienna, Austria) using the data.table (1.18.2.1), lme4 (2.0-1), emmeans (2.0.2), splines (4.5.3), and ggplot2 (4.0.2) packages.

### 2.8. Ethics

All data provided by JMDC, Inc., were fully deidentified and anonymized before analysis. Because the dataset was anonymized, individual opt-out was not feasible; instead, this was disclosed through a publicly available information notice in accordance with institutional requirements. The study protocol was reviewed and approved by the Institutional Review Board of (institutional name) (approval no. HM25-712; approval date: 12 April 2026). This was a retrospective cohort study using pre-existing health check-up records collected between 2005 and 2025, and ethical approval for secondary analysis of these existing data was obtained on 12 April 2026.

## 3. Results

### 3.1. Baseline Characteristics of the Participants in This Study

As shown in Table 1, participants were first classified into three mutually exclusive groups according to their BMI records in their twenties: an underweight-history group, defined as those who had at least one health check-up with BMI < 18.5 kg/m^2^; an obesity-history group, defined as those who had at least one health check-up with BMI ≥ 25.0 kg/m^2^; and a normal-weight-only group, defined as those whose BMI remained within the normal range throughout their twenties. I then compared the subsequent BMI trajectories across these three groups. Overall, 57,039 women (31.0%) and 50,508 men (13.3%) were classified into the underweight-history group, 101,526 women (55.1%) and 216,770 men (57.1%) into the normal-weight-only group, and 25,697 women (13.9%) and 112,658 men (29.7%) into the obesity-history group. The mean baseline age was 24 years. Mean BMI clearly differed across the three groups, ranging from 18.0 kg/m^2^ in women and 17.9 kg/m^2^ in men in the underweight-history group to 26.6 kg/m^2^ in both women and men in the obesity-history group. The mean observation period was 6–7 years (Table 1).

Self-reported eating speed also differed across the three groups. Fast eating was most prevalent in the obesity-history group and least prevalent in the underweight-history group, whereas slow eating showed the opposite pattern. Specifically, fast eating was reported by 17.5% of women and 19.6% of men in the underweight-history group, 24.0% of women and 32.7% of men in the normal-weight-only group, and 29.4% of women and 48.2% of men in the obesity-history group (Table 1). In contrast, slow eating was most common in the underweight-history group, where it was reported by 24.9% of women and 21.6% of men, and least common in the obesity-history group, where it was reported by 13.4% of women and 6.2% of men. Breakfast skipping, late-evening meals, and irregular meal timing showed smaller between-group differences than self-reported eating speed (Table 1).

Further subdivision into persistence-based nine groups is summarized in Supplementary Table S1. Within the underweight-history and obesity-history groups, the 75–100% persistence category was the most common, particularly in the obesity-history group.

**Table 1:** Baseline characteristics of participants according to weight-history group in their twenties.

Variable	History of UW	NW Only in 20s	History of OB
Participants,	F: 57,039 (31.0)	F: 101,526 (55.1)	F: 25,697 (13.9)
n (% within sex)	M: 50,508 (13.3)	M: 216,770 (57.1)	M: 112,658 (29.7)
Age at baseline,	F: 24.2 (2.7)	F: 24.9 (2.8)	F: 24.4 (2.8)
mean (SD), years	M: 23.7 (2.8)	M: 24.9 (2.9)	M: 24.4 (2.9)
BMI at baseline,	F: 18.0 (1.2)	F: 20.9 (1.5)	F: 26.6 (3.9)
mean (SD), kg/m^2^	M: 17.9 (1.1)	M: 21.3 (1.5)	M: 26.6 (3.7)
Observed age span,	F: 6.0 [5.0, 8.0]	F: 6.0 [5.0, 8.0]	F: 6.0 [5.0, 8.0]
median [Q1, Q3], years	M: 6.0 [5.0, 9.0]	M: 7.0 [5.0, 9.0]	M: 7.0 [5.0, 9.0]
Breakfast skipping,	F: 28.7 (13,252/46,191)	F: 30.0 (24,548/81,851)	F: 36.3 (7501/20,684)
% (n/N)	M: 39.7 (14,579/36,690)	M: 37.9 (57,543/151,901)	M: 40.0 (32,109/80,254)
Late-evening meals,	F: 33.5 (15,547/46,436)	F: 34.4 (28,314/82,415)	F: 36.5 (7590/20,821)
% (n/N)	M: 39.5 (14,732/37,319)	M: 40.8 (63,190/154,805)	M: 41.2 (33,750/81,909)
Irregular meal timing,	F: 13.9 (6381/46,059)	F: 14.6 (11,901/81,627)	F: 17.7 (3654/20,624)
% (n/N)	M: 20.2 (7352/36,456)	M: 19.7 (29,725/150,537)	M: 20.9 (16,662/79,778)
Fast eating,	F: 17.5 (8050/45,869)	F: 24.0 (19,527/81,396)	F: 29.4 (6044/20,589)
% (n/N)	M: 19.6 (7112/36,326)	M: 32.7 (49,043/149,908)	M: 48.2 (38,172/79,249)
Slow eating,	F: 24.9 (11,439/45,869)	F: 17.3 (14,050/81,396)	F: 13.4 (2750/20,589)
% (n/N)	M: 21.6 (7836/36,326)	M: 11.7 (17,597/149,908)	M: 6.2 (4894/79,249)

UW indicates underweight-history group; NW indicates normal weight-history group; and OB indicates obesity-history group. The upper section represents females (F), and the lower section represents males (M). The percentages of participants are shown within each sex. Baseline was defined as each participant’s first health check record between the ages of 20 and 29 years. The observed age span was calculated as the maximum examination age minus the minimum examination age. Irregular meal timing was defined as both breakfast skipping and late-evening meals.

### 3.2. Cross-Sectional BMI Categories at Age 35 Years Across Weight-History Groups

**Figure 2:**
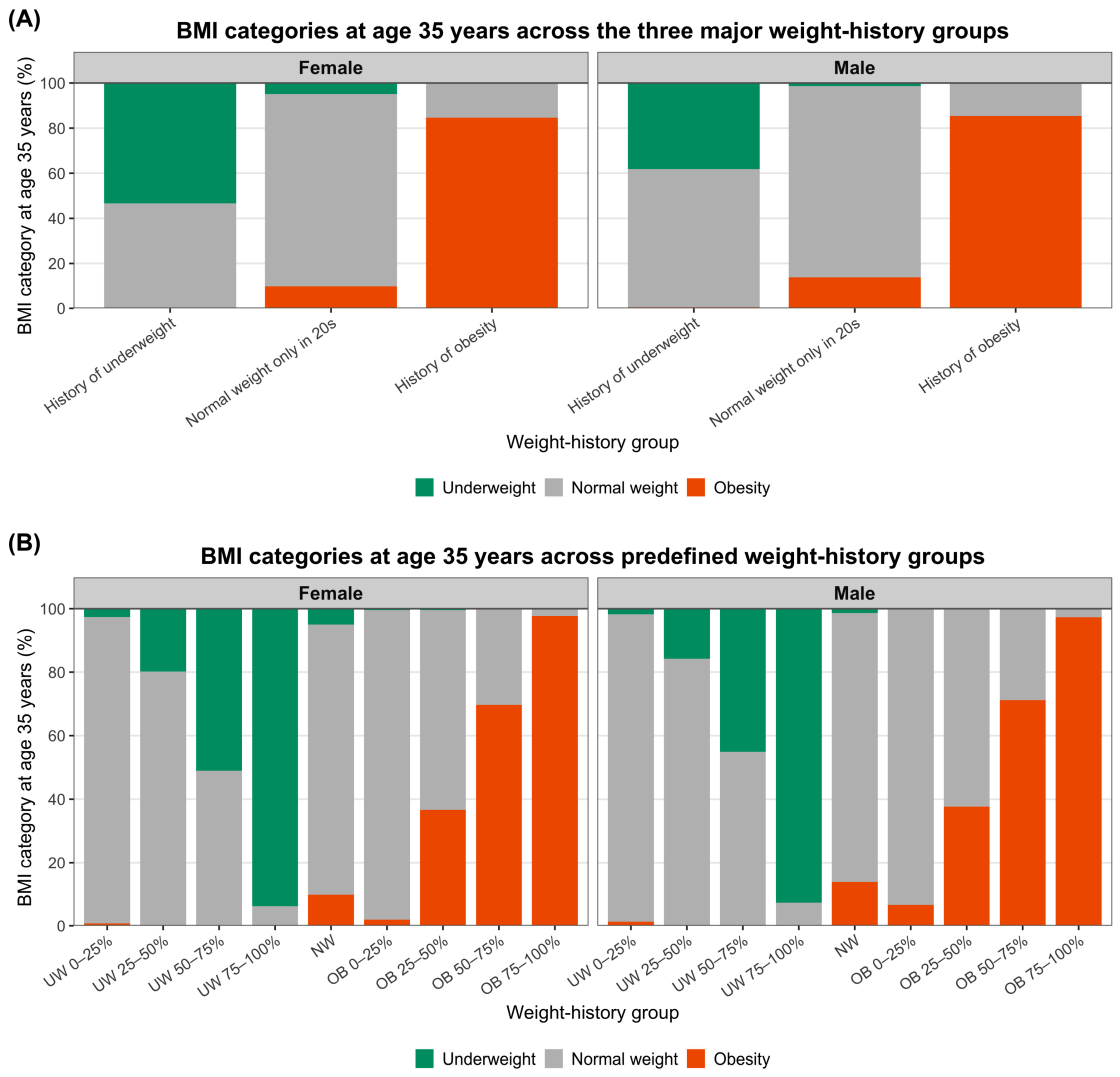
Cross-sectional BMI categories at age 35 years across three and nine weight-history groups.

To further illustrate why a single BMI category at one follow-up age could not fully capture long-term weight-history groups, BMI categories at age 35 years were examined according to the predefined weight-history groups (Figure 2; Supplementary Table S2). In the UW 0–25% group, 96.6% of women and 96.9% of men were classified as normal weight at age 35 years, despite belonging to the underweight-history group. Similarly, in the OB 0–25% group, 97.6% of women and 93.3% of men were classified as normal weight at age 35 years, despite belonging to the obesity-history group. In contrast, in the most persistent groups, 93.8% of women and 92.6% of men in the UW 75–100% group were underweight, whereas 97.7% of women and 97.4% of men in the OB 75–100% group were obese. These findings indicate that a single cross-sectional BMI category at follow-up did not adequately reflect the cumulative duration or persistence of underweight and obesity.

BMI categories at age 35 years are shown according to the predefined three major weight-history groups (A) and nine weight-history groups (B), separately for women and men. In panel A, participants were classified into a history of underweight, normal weight only in the twenties, and a history of obesity. In panel B, the underweight-history and obesity-history groups were further stratified according to the corresponding underweight or obesity proportion (0–25%, 25–50%, 50–75%, and 75–100%), together with the normal-weight-only group. Bars indicate the proportion of participants with an available BMI measurement at age 35 years who were classified as underweight (BMI < 18.5 kg/m^2^), normal weight (BMI 18.5–24.9 kg/m^2^), or obesity (BMI ≥ 25.0 kg/m^2^). This figure illustrates that a single cross-sectional BMI category at age 35 years does not fully capture long-term weight-history groups.

### 3.3. Body-Size Trajectories Across Three and Nine Weight-History Groups

Estimated marginal means at ages 25, 35, and 45 years were calculated using linear mixed-effects models in which age was modeled using natural cubic spline terms, and participant-specific random intercepts and random slopes for age were included to account for within-person correlation.

**Figure 3:**
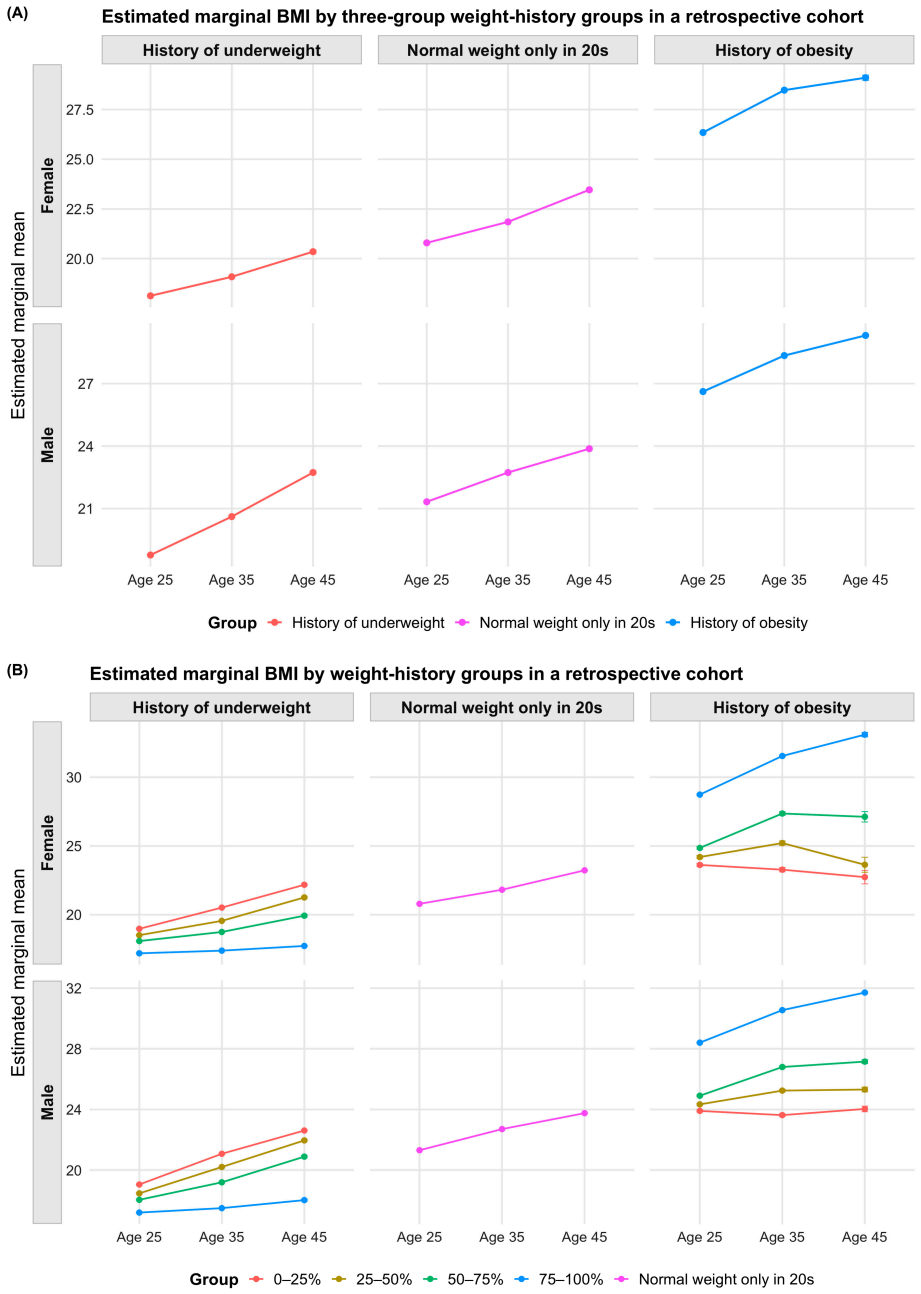
Estimated marginal BMI trajectories across three-group and nine-group weight-history groups in a retrospective cohort.

I first compared BMI trajectories across the three broad weight-history groups. In both women and men, the underweight-history, normal-weight-only, and obesity-history groups showed clearly distinct BMI trajectories over follow-up, and the interaction between weight-history group and age was statistically significant in both sexes (*p* for interaction < 0.001; Figure 3A).

I further subdivided the underweight-history and obesity-history groups according to their underweight or obesity proportion during the entire observation period, generating nine weight-history groups, to examine whether subsequent BMI trajectories differed according to the persistence of weight status (Figure 3B, Table 2). In both women and men, BMI trajectories differed clearly across these nine weight-history groups, with significant interactions between weight-history group and age in all analyses (all *p* for interaction < 0.001).

In both men and women, BMI trajectories differed clearly across the nine weight-history groups (Figure 2B, Table 2). Among participants with a history of underweight, the 75–100% group remained substantially leaner than the other underweight-history groups throughout follow-up. In women, BMI in this group increased only modestly from 17.20 (95% CI, 17.19–17.21) at age 25 to 18.12 (95% CI, 18.03–18.21) at age 45, whereas in the 0–25% group, it increased from 18.98 (95% CI, 18.96–18.99) to 22.54 (95% CI, 22.42–22.66) (Figure 3B, Table 2). A similar pattern was observed in men, whose BMI in the 75–100% underweight group changed from 17.20 (95% CI, 17.19–17.22) at age 25 to 18.03 (95% CI, 17.97–18.09) at age 45 (Figure 3B, Table 2).

Among participants with a history of obesity, the 75–100% group consistently showed the highest BMI across follow-up in both sexes. In women, BMI in this group increased from 28.72 (95% CI, 28.66–28.78) at age 25 to 32.86 (95% CI, 32.58–33.14) at age 45, while the corresponding values in the 0–25% group remained lower, changing from 23.92 (95% CI, 23.88–23.96) to 22.77 (95% CI, 22.57–22.97) (Figure 3B, Table 2). Men showed the same graded pattern, with BMI in the 75–100% obesity group increasing from 28.31 (95% CI, 28.28–28.35) at age 25 to 31.84 (95% CI, 31.72–31.96) at age 45 (Figure 3B and Table 2). These findings indicate that the proportion of young adults who spent time in underweight or obesity states was reflected in distinct mid-adult body-size trajectories.

The estimated marginal means of BMI at ages 25, 35, and 45 years were calculated for the three broad weight-history groups (A) and the nine weight-history groups (B), comprising four underweight-proportion groups, the normal-weight-only group, and four obesity-proportion groups. The y-axis shows the estimated marginal BMI, and the x-axis shows age. Trajectories were estimated using linear mixed-effects models with natural cubic spline terms for age and participant-specific random intercepts and random slopes for age. The models included interaction terms between weight-history group and age to allow trajectory patterns to differ across groups. Error bars indicate 95% confidence intervals. Underweight-proportion and obesity-proportion groups were defined according to proportion of health checkups during the entire observation period at which participants were classified as underweight or obese, respectively (0–25%, 25–50%, 50–75%, and 75–100%).

**Table 2:** Estimated marginal mean BMI (95% confidence interval) at ages 25, 35, and 45 years according to weight-history group in this retrospective cohort.

Weight-History Group	Female Age 25	Female Age 35	Female Age 45	Male Age 25	Male Age 35	Male Age 45
UW 0–25%	18.97 (18.96–18.98)	20.52 (20.49–20.54)	22.18 (22.11–22.24)	19.06 (19.04–19.07)	21.08 (21.06–21.10)	22.61 (22.57–22.66)
UW 25–50%	18.51 (18.49–18.52)	19.55 (19.53–19.57)	21.25 (21.17–21.33)	18.47 (18.45–18.49)	20.21 (20.19–20.23)	21.96 (21.90–22.02)
UW 50–75%	18.08 (18.06–18.09)	18.74 (18.72–18.76)	19.93 (19.84–20.01)	18.05 (18.03–18.07)	19.20 (19.17–19.23)	20.89 (20.82–20.97)
UW 75–100%	17.19 (17.18–17.20)	17.38 (17.37–17.40)	17.72 (17.67–17.78)	17.20 (17.19–17.22)	17.50 (17.48–17.52)	18.03 (17.97–18.09)
NW only	20.79 (20.78–20.80)	21.82 (21.80–21.83)	23.23 (23.19–23.26)	21.31 (21.31–21.32)	22.71 (22.70–22.72)	23.76 (23.74–23.77)
OB 0–25%	23.62 (23.51–23.72)	23.27 (23.12–23.43)	22.73 (22.24–23.22)	23.91 (23.84–23.97)	23.63 (23.54–23.72)	24.04 (23.85–24.22)
OB 25–50%	24.19 (24.09–24.30)	25.21 (25.05–25.36)	23.63 (23.09–24.18)	24.34 (24.28–24.40)	25.25 (25.17–25.32)	25.32 (25.16–25.47)
OB 50–75%	24.85 (24.76–24.95)	27.36 (27.23–27.49)	27.12 (26.74–27.50)	24.91 (24.86–24.96)	26.80 (26.74–26.87)	27.16 (27.04–27.28)
OB 75–100%	28.73 (28.68–28.79)	31.54 (31.48–31.61)	33.10 (32.94–33.26)	28.41 (28.38–28.43)	30.55 (30.52–30.58)	31.71 (31.65–31.76)

Abbreviations: BMI, body mass index; UW, underweight; NW, normal weight; OB, obesity. Estimated marginal means were derived from linear mixed-effects models with restricted cubic spline terms for age and participant-specific random intercepts and random slopes for age. Weight-history groups were defined from BMI records during the participants’ twenties. Participants were classified into nine groups: four underweight-duration groups, one normal-weight-only group, and four obesity-duration groups. The underweight-duration and obesity-duration groups were defined according to the proportion of health check-ups during the participants’ twenties at which participants were classified as underweight or obese, respectively (0–25%, 25–50%, 50–75%, and 75–100%). Estimated marginal means were derived from linear mixed-effects models with restricted cubic spline terms for age and participant-specific random intercepts and random slopes for age.

### 3.4. Self-Reported Eating Speed and Weight-History Groups

**Figure 4:**
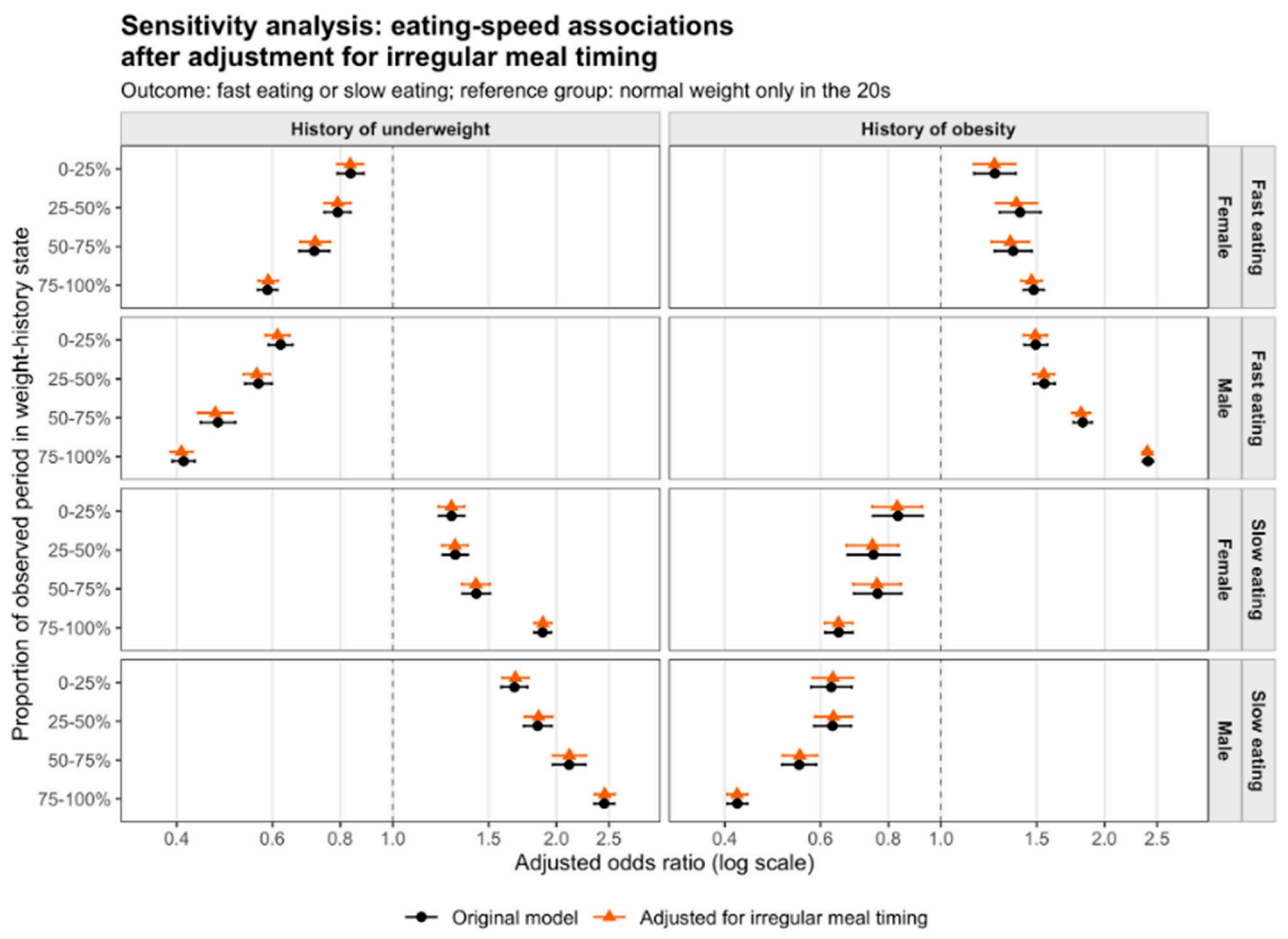
Associations of self-reported fast and slow eating with weight-history groups, before and after additional adjustment for irregular meal timing.

Participants who reported fast eating at their first health check-up tended to spend a greater proportion of time in the obesity range, whereas those who reported slow eating tended to spend a greater proportion of time in the underweight range. Therefore, I examined the associations between self-reported eating speed and weight-history groups. Fast eating has long been linked to obesity, and our results extended this association to longitudinal weight-history groups. In women, the odds of fast eating decreased progressively as the underweight proportion increased, whereas the odds of slow eating increased (Figure 3 and Table 3). The same overall pattern was observed in men (Figure 4 and Table 3). Thus, persistent underweight was characterized by lower odds of fast eating and higher odds of slow eating, whereas persistent obesity showed the opposite pattern.

Adjusted odds ratios for fast and slow eating according to weight-history group are shown separately for women and men. Odds ratios were estimated using logistic regression models adjusted for age, sleep, exercise habits, physical activity, and walking speed. Estimates are shown for the original adjusted model and for a further adjusted model that additionally included irregular meal timing. The normal-weight-only group during the participants’ twenties was used as the reference group. Error bars indicate 95% confidence intervals. The close overlap between the two estimates indicates that the associations between self-reported eating speed and weight-history groups were not materially attenuated by additional adjustment for irregular meal timing.

In sensitivity analyses, additional adjustment for irregular meal timing did not materially alter these associations, indicating that the association between self-reported eating speed and weight-history group was largely independent of meal-timing behaviors (Figure 4 and Table 3). Notably, the association for fast eating was stronger in men, whereas the association for slow eating was stronger in women (Figure 4 and Table 3).

**Table 3:** Associations of self-reported fast and slow eating with weight-history groups, before and after additional adjustment for irregular meal timing.

		Fast Eating		Slow Eating	
Sex	Group	Unadjusted OR (95% CI)	Adjusted OR (95% CI)	Unadjusted OR (95% CI)	Adjusted OR (95% CI)
Female	UW 0–25%	0.84 (0.79–0.88)	0.83 (0.79–0.88)	1.28 (1.21–1.35)	1.28 (1.21–1.35)
	UW 25–50%	0.79 (0.75–0.84)	0.79 (0.75–0.84)	1.30 (1.23–1.37)	1.30 (1.23–1.37)
	UW 50–75%	0.72 (0.67–0.76)	0.72 (0.67–0.77)	1.42 (1.34–1.51)	1.42 (1.34–1.51)
	UW 75–100%	0.59 (0.56–0.61)	0.59 (0.57–0.61)	1.89 (1.82–1.95)	1.89 (1.82–1.96)
	OB 0–25%	1.26 (1.15–1.37)	1.25 (1.15–1.37)	0.83 (0.75–0.93)	0.83 (0.75–0.92)
	OB 25–50%	1.40 (1.28–1.53)	1.38 (1.26–1.50)	0.75 (0.67–0.84)	0.75 (0.67–0.84)
	OB 50–75%	1.36 (1.25–1.47)	1.34 (1.24–1.45)	0.76 (0.69–0.85)	0.76 (0.69–0.84)
	OB 75–100%	1.48 (1.42–1.55)	1.47 (1.40–1.53)	0.65 (0.61–0.69)	0.65 (0.61–0.69)
Male	UW 0–25%	0.62 (0.59–0.65)	0.61 (0.58–0.65)	1.67 (1.58–1.77)	1.68 (1.59–1.78)
	UW 25–50%	0.57 (0.53–0.60)	0.56 (0.53–0.59)	1.85 (1.74–1.96)	1.85 (1.75–1.97)
	UW 50–75%	0.48 (0.44–0.51)	0.47 (0.44–0.51)	2.11 (1.97–2.26)	2.11 (1.97–2.27)
	UW 75–100%	0.41 (0.39–0.43)	0.41 (0.39–0.43)	2.45 (2.35–2.56)	2.45 (2.35–2.56)
	OB 0–25%	1.49 (1.42–1.57)	1.49 (1.42–1.57)	0.63 (0.58–0.68)	0.63 (0.58–0.69)
	OB 25–50%	1.55 (1.48–1.62)	1.55 (1.48–1.62)	0.63 (0.58–0.68)	0.63 (0.59–0.69)
	OB 50–75%	1.82 (1.75–1.89)	1.81 (1.74–1.88)	0.55 (0.51–0.59)	0.55 (0.51–0.59)
	OB 75–100%	2.40 (2.35–2.46)	2.40 (2.35–2.45)	0.42 (0.40–0.44)	0.42 (0.40–0.44)

Reference group: normal weight only during the participants’ twenties. UW indicates underweight-history group; OB indicates obesity-history group. The original adjusted model was adjusted for age, sleep, exercise habits, physical activity, and walking speed. The further adjusted model included additional adjustment for irregular meal timing. All *p* values were <0.001.

**Figure 5:**
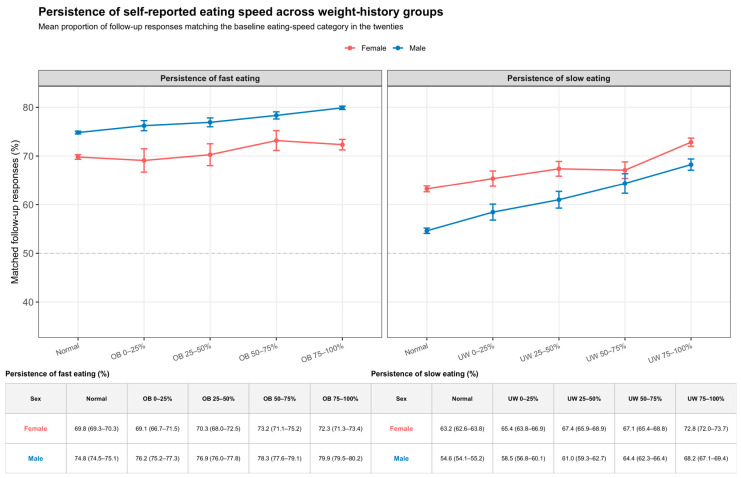
Persistence of self-reported eating speed across weight-history groups.

As a supplementary analysis, I examined the persistence of self-reported eating speed during follow-up according to weight-history group (Figure 5). The proportion of follow-up responses matching the baseline category tended to increase with increasing persistence of obesity for fast eating and with increasing persistence of underweight for slow eating (Figure 5). These patterns were observed in both women and men.

Taken together, these findings suggest that self-reported eating speed is consistently associated with long-term weight-history groups.

Percentages indicate the proportion of subsequent non-missing self-reported eating-speed responses that matched the baseline self-reported eating-speed category reported at the first health check-up in the twenties. The left panel shows the persistence of fast eating across the normal-weight-only and obesity-related weight-history groups, and the right panel shows the persistence of slow eating across the normal-weight-only and underweight-related weight-history groups. The results are shown separately for women and men. Data are represented as mean (95%CI).

## 4. Discussion

The present study extends previous research in two important ways. First, rather than focusing only on obesity or BMI at a single time point, I examined longitudinal weight-history groups representing both underweight- and obesity-related weight-history groups. Second, I evaluated self-reported eating speed in relation to these long-term groups, thereby assessing its association with both extremes of body weight.

In our longitudinal cohort, approximately 40% of participants with a history of underweight and 60% of those with a history of obesity belonged to the most persistent groups, defined as spending 75–100% of follow-up in the respective weight state. Self-reported eating speed showed a consistent association with weight-history group in both sexes: as the underweight proportion increased, the odds of slow eating increased and the odds of fast eating decreased, whereas a mirror-image pattern was observed with increasing obesity proportion. Slow eating was particularly common among participants with persistent underweight. Taken together, these findings suggest that self-reported eating speed may be associated with long-term weight-history groups at both extremes of body weight.

A key implication of the present study is the importance of viewing body weight as a longitudinal trajectory rather than as a single cross-sectional state. A single BMI measurement captures only one moment in time and cannot distinguish between individuals who remain in the same weight state and those who transition between underweight, normal weight, and obesity over time [26]. This distinction is clinically relevant because the cumulative burden of abnormal weight states appears to be relevant to later health outcomes. Previous cohort studies have shown that a longer duration of obesity is associated with worse cardiometabolic risk profiles, whereas cumulative exposure to underweight is associated with a higher risk of hip fracture [15,28,29,30,31]. In this context, our weight-history groups provide a more informative framework than BMI measured at a single time point alone for understanding long-term body-size regulation and its behavioral correlates.

Consistent with this perspective, persistent underweight remained associated with low BMI through age 45, whereas persistent obesity was associated with persistently high and increasing BMI over time. These results are also in line with our previous findings on underweight women [26]. Notably, the group with the lowest baseline BMI was the persistent underweight group, whereas the group with the highest baseline BMI in the obesity category was the persistent obesity group. Although future trajectories cannot be predicted from BMI alone, because individuals may transition between underweight and normal weight over time, baseline BMI appears to be an important baseline characteristic associated with subsequent body-weight trajectory [26]. Taken together, these findings indicate that the persistence of underweight and obesity in young adulthood is strongly reflected in subsequent body-size trajectories.

Self-reported eating speed showed a robust and consistent association with weight-history groups. As the duration of underweight increased, the odds of slow eating increased and the odds of fast eating decreased, whereas the opposite pattern was observed with increasing obesity duration. These associations were largely unchanged after additional adjustment for irregular meal timing, suggesting that the observed relationship between self-reported eating speed and weight-history groups was not substantially explained by meal-timing behaviors. Previous studies have focused primarily on obesity and metabolic syndrome [7,8,9,10,11,12,13,14,15,16,41]. In a systematic review and meta-analysis, Yuan et al. reported that, compared with slower eating, faster eating was associated with higher risks of metabolic syndrome, central obesity, elevated blood pressure, low HDL cholesterol, elevated triglycerides, and elevated fasting plasma glucose [11,12,13,14,15]. Similarly, Sasaki et al. reported that self-reported eating speed was positively associated with BMI in 18-year-old Japanese women [8], and Otsuka et al. reported that BMI increased progressively with faster self-reported eating speed in both men and women [9]. Moreover, eating speed may reflect not only chewing duration and meal pace, but also broader eating patterns and dietary habits [42,43,44,45,46]. In our previous work, I found that even with similar calorie content, fast-food meals were consumed more quickly than boxed meals, suggesting that food choice itself may be related to eating speed [43]. Thus, food selection may be one factor related to the observed association between faster eating and obesity-related groups.

In contrast, slower eating may prolong mealtime and promote satiation before adequate energy intake is achieved, which could contribute to persistent underweight. Slower eating has been proposed to enhance satiety-related responses, potentially involving gut-derived hormones such as cholecystokinin (CCK), glucagon-like peptide-1 (GLP-1), and peptide YY (PYY), which promote postprandial fullness, together with lower ghrelin responses, which may reduce hunger after meals [47,48]. Leptin, a longer-term regulator of energy balance derived mainly from adipose tissue, may also be relevant to these satiety-related pathways by modulating central appetite regulation [48]. Longer oral processing and more thorough chewing may further enhance central satiety signaling and earlier meal termination [49]. In addition, longer meal duration and slower oral processing have been suggested to influence postprandial energy expenditure, including diet-induced thermogenesis [50,51]. These interpretations remain speculative, but they may help contextualize the bidirectional associations observed in the present study. It should also be noted that many previous studies, including the present one, relied on self-reported eating speed rather than objective measures of eating behavior. Therefore, these associations may reflect individuals’ subjective perception of their own eating speed rather than objectively measured eating behavior, such as eating rate during a standardized test meal or meal duration in daily life.

Importantly, the association between self-reported eating speed and weight-history groups was bidirectional rather than one-sided. Persistent underweight and persistent obesity showed a mirror-image pattern, with slower eating being more common in underweight-related groups and faster eating being more common in obesity-related groups. An additional finding of the present study was the persistence of self-reported eating speed over time. Participants with persistent obesity-related groups tended to continue perceiving themselves as fast eaters, whereas those with persistent underweight-related groups tended to continue perceiving themselves as slow eaters. These findings suggest that self-reported eating speed may be relatively persistent over time. An important next step will be to examine whether changes in self-reported eating speed over time are associated with subsequent body-weight change.

This study has several limitations. First, the cohort was derived from a Japanese health insurance database and may not fully represent the general Japanese population, although the overall pattern was consistent with that observed in our previous single-center longitudinal study. Second, eating habits and eating speed were based on self-reports and are therefore subject to misclassification. In particular, self-reported eating speed reflects participants’ perception of their own eating speed rather than an objective measurement of actual eating rate. Previous studies have also shown that self-reported speed of eating does not necessarily correspond to objectively measured eating rate [52,53]. Thus, although this variable may not fully capture true eating speed, it may still be meaningful as an indicator of subjective awareness of eating style. In future studies, I plan to directly compare self-reported eating speed, objectively measured eating speed during a standardized test meal, and usual meal duration in daily life. Third, the health check-up database did not include several potentially important confounders, such as smoking, alcohol consumption, socioeconomic status, occupational category, and marital status; therefore, residual confounding could not be excluded. Fourth, because this was an observational study, causal relationships cannot be established. Fifth, although the database covered a 20-year period, the mean duration of follow-up was approximately 6 years, and trajectories beyond mid-adulthood could not be evaluated. Sixth, there is a potential for cohort or period effects. Because participants entered the database in different calendar years, changes in the social and food environment between 2005 and 2025 may have influenced the observed trajectories. Thus, age-related patterns could not be fully disentangled from calendar-period or birth-cohort effects. Further longitudinal studies with objective assessments of eating behaviors and longer follow-up into older adulthood are warranted.

## 5. Conclusions

Taken together, the present study provides two main insights. First, even among individuals with a history of underweight or obesity in their twenties, subsequent BMI trajectories differed substantially according to the corresponding underweight or obesity proportion, indicating that weight-history groups capture important heterogeneity in long-term body weight patterns. Second, among the eating behaviors examined, self-reported eating speed showed a more consistent association with long-term weight-history groups than irregular meal timing. Slower eating was more frequently observed in persistent underweight-related groups, whereas faster eating was more frequently observed in persistent obesity-related groups. These findings suggest that self-reported eating speed may be associated with subsequent long-term weight-history groups.

## Data Availability

The data used in this study were obtained under a data-use agreement with JMDC Inc. and cannot be shared by the authors with third parties because of contractual restrictions. The data may be purchased directly from JMDC Inc., subject to its data-access procedures, approval, and applicable fees.

## Supplementary Materials

The following supporting information can be downloaded at https://www.mdpi.com/article/10.3390/nu18152412/s1, Table S1: Background data of the participants in this study (nine groups); Table S2. Cross-sectional BMI categories at ages 30, 35, and 40 years according to three and nine weight-history groups.
