# Supplementary material for: Self-Reported Eating Speed Is Dose-Dependently and Bidirectionally Associated with Long-Term Underweight- and Obesity-Related Weight-History Groups
Source: Nutrients. 2026 Jul 24;18(15):2412. doi: 10.3390/nu18152412 (PMC13468294; doi:10.3390/nu18152412)
Supplement: Supplementary file 1 [file nutrients-18-02412-s001.zip › nutrients-4403456-supplementary.pdf]

Supplementary Results.

Supplementary Table 1. Background data of the participants in this study (nine groups)

Among those who were underweight at least once in their twenties, 44.7% of women and 36.4% of men remained underweight for 75–100% of the period (Supplemantary table 1). Additionally, among those who were obese at least once in their twenties, 57.3% of women and 62.4% of men remained overweight for 75–100% of the period (Supplemantary table 1). Additionally, the BMI of individuals who were underweight 75–100% of the time was 17.3 (0.9) for females and 17.2 (0.9) for males, which was the lowest in this group. The BMI of individuals who were underweight for 75–100% of the time was 17.3 (0.9) for females and 17.2 (0.9) for males, which was the lowest among the underweight group (Supplemantary table 1). On the other hand, the BMI of individuals who were obese for 75–100% of the time was 28.5 (3.9) for females and 28.2 (3.6) for males, which was the highest among the underweight group (Supplemantary table 1).

Supplementary Table S1. Background data of the participants in this study (nine group)

A) Underweight-duration groups

Variable	UW 0-25%	UW 25-50%	UW 50-75%	UW 75-100%
Participants, n (% within phenotype)	F: 11,474 (20.1)<br>M: 13,917 (27.6)	F: 11,337 (19.9)<br>M: 10,950 (21.7)	F: 8,752 (15.3)<br>M: 7,231 (14.3)	F: 25,476 (44.7)<br>M: 18,410 (36.4)
Age at baseline, mean (SD), years	F: 23.9 (2.6)<br>M: 23.6 (2.7)	F: 24.1 (2.6)<br>M: 23.7 (2.8)	F: 24.2 (2.7)<br>M: 23.6 (2.9)	F: 24.4 (2.7)<br>M: 23.8 (2.9)
BMI at baseline, mean (SD), kg/m2	F: 19.0 (1.0)<br>M: 18.7 (1.0)	F: 18.6 (0.9)<br>M: 18.3 (0.9)	F: 18.2 (0.9)<br>M: 18.0 (0.8)	F: 17.3 (0.9)<br>M: 17.2 (0.9)
Observed age span, median [Q1, Q3], years	F: 6.0 [5.0, 8.0]<br>M: 7.0 [5.0, 10.0]	F: 6.0 [5.0, 8.0]<br>M: 6.0 [5.0, 9.0]	F: 6.0 [5.0, 8.0]<br>M: 6.0 [5.0, 9.0]	F: 6.0 [4.0, 7.0]<br>M: 6.0 [4.0, 8.0]
Breakfast skipping, % (n/N)	F: 29.8 (2,714/9,102)<br>M: 40.6 (3,897/9,605)	F: 29.6 (2,717/9,172)<br>M: 40.2 (3,172/7,899)	F: 28.6 (2,018/7,064)<br>M: 38.0 (1,958/5,146)	F: 27.8 (5,803/20,853)<br>M: 39.5 (5,552/14,040)
Late-evening meals, % (n/N)	F: 33.7 (3,081/9,140)<br>M: 40.1 (3,911/9,745)	F: 34.4 (3,177/9,234)<br>M: 39.5 (3,179/8,044)	F: 33.2 (2,356/7,094)<br>M: 39.7 (2,074/5,230)	F: 33.1 (6,933/20,968)<br>M: 38.9 (5,568/14,300)
Irregular meal timing, % (n/N)	F: 14.5 (1,318/9,068)<br>M: 21.1 (2,002/9,505)	F: 14.3 (1,306/9,147)<br>M: 20.2 (1,585/7,847)	F: 13.6 (960/7,044)<br>M: 19.2 (982/5,115)	F: 13.4 (2,797/20,800)<br>M: 19.9 (2,783/13,989)
Fast eating, % (n/N)	F: 20.0 (1,803/9,012)<br>M: 22.8 (2,154/9,460)	F: 19.4 (1,771/9,130)<br>M: 21.3 (1,674/7,841)	F: 18.1 (1,266/6,994)<br>M: 18.6 (947/5,084)	F: 15.5 (3,210/20,733)<br>M: 16.8 (2,337/13,941)

Slow eating, % (n/N)	F: 21.5 (1,941/9,012)<br>M: 18.5 (1,750/9,460)	F: 21.7 (1,984/9,130)<br>M: 19.7 (1,548/7,841)	F: 23.2 (1,626/6,994)<br>M: 22.0 (1,119/5,084)	F: 28.4 (5,888/20,733)<br>M: 24.5 (3,419/13,941)

(B) Obesity-duration groups

Variable	OB 0-25%	OB 25-50%	OB 50-75%	OB 75-100%
Participants, n (% within phenotype)	F: 3,495 (13.6)<br>M: 10,757 (9.5)	F: 3,397 (13.2)<br>M: 13,407 (11.9)	F: 4,086 (15.9)<br>M: 18,157 (16.1)	F: 14,719 (57.3)<br>M: 70,337 (62.4)
Age at baseline, mean (SD), years	F: 23.1 (2.4)<br>M: 23.0 (2.5)	F: 23.4 (2.5)<br>M: 23.4 (2.6)	F: 24.1 (2.6)<br>M: 23.9 (2.7)	F: 25.0 (2.9)<br>M: 25.0 (2.9)
BMI at baseline, mean (SD), kg/m2	F: 23.4 (1.9)<br>M: 23.4 (1.7)	F: 24.0 (2.0)<br>M: 23.8 (1.8)	F: 24.6 (2.0)<br>M: 24.5 (1.8)	F: 28.5 (3.9)<br>M: 28.2 (3.6)
Observed age span, median [Q1, Q3], years	F: 6.0 [4.0, 7.0]<br>M: 6.0 [5.0, 8.0]	F: 6.0 [5.0, 7.0]<br>M: 6.0 [5.0, 8.0]	F: 6.0 [5.0, 8.0]<br>M: 7.0 [5.0, 9.0]	F: 6.0 [5.0, 8.0]<br>M: 7.0 [5.0, 9.0]
Breakfast skipping, % (n/N)	F: 31.0 (864/2,787)<br>M: 37.5 (2,923/7,793)	F: 35.2 (963/2,737)<br>M: 38.3 (3,663/9,575)	F: 36.9 (1,209/3,273)<br>M: 40.2 (5,117/12,742)	F: 37.6 (4,465/11,887)<br>M: 40.7 (20,406/50,144)
Late-evening meals, % (n/N)	F: 34.9 (982/2,810)<br>M: 38.1 (3,050/8,010)	F: 37.4 (1,029/2,754)<br>M: 38.9 (3,804/9,787)	F: 38.0 (1,251/3,296)<br>M: 41.1 (5,348/13,026)	F: 36.2 (4,328/11,961)<br>M: 42.2 (21,548/51,086)
Irregular meal timing, % (n/N)	F: 15.4 (429/2,781)<br>M: 18.5 (1,438/7,768)	F: 18.1 (495/2,732)<br>M: 18.8 (1,793/9,538)	F: 18.3 (597/3,262)<br>M: 20.9 (2,647/12,674)	F: 18.0 (2,133/11,849)<br>M: 21.7 (10,784/49,798)
Fast eating, % (n/N)	F: 26.1 (726/2,777)<br>M: 39.7 (3,079/7,747)	F: 28.3 (771/2,723)<br>M: 41.3 (3,922/9,506)	F: 28.5 (932/3,267)<br>M: 45.2 (5,709/12,627)	F: 30.6 (3,615/11,822)<br>M: 51.6 (25,462/49,369)
Slow eating, % (n/N)	F: 15.7 (437/2,777)<br>M: 7.8 (608/7,747)	F: 14.0 (382/2,723)<br>M: 7.8 (743/9,506)	F: 14.3 (466/3,267)<br>M: 6.9 (870/12,627)	F: 12.4 (1,465/11,822)<br>M: 5.4 (2,673/49,369)

UW indicates underweight-history group; NW indicates normal weight-history group; and OB in-dicates obesity-history group. The upper section represents females (F), and the lower section rep-resents males (M). The percentages of participants are shown within each sex. Baseline was defined as each participant’s first health check record between the ages of 20 and 29 years. The observed age span was calculated as the maximum examination age minus the minimum examination age. Irregular meal timing was defined as both breakfast skipping and late-evening meals.

Supplementary Table S2. Cross-sectional BMI categories at ages 30, 35, and 40 years according to three and nine weight-history groups.

(A)

Age	Sex	Weight-history group	N with BMI at this age	UW	NW	Ob
**35**	Female	History of underweight	8790	53.4% (4690)	46.4% (4079)	0.2% (21)
**35**	Female	Normal weight only in 20s	21501	4.9% (1059)	85.2% (18329)	9.8% (2113)
**35**	Female	History of obesity	4279	0.0% (2)	15.4% (657)	84.6% (3620)
**35**	Male	History of underweight	10257	38.1% (3909)	61.4% (6295)	0.5% (53)
**35**	Male	Normal weight only in 20s	66198	1.4% (894)	84.8% (56151)	13.8% (9153)
**35**	Male	History of obesity	27163	0.0% (1)	14.6% (3977)	85.4% (23185)

(**B**)

Age	Sex	Weight-history group	N with BMI at this age	UW	NW	Ob
**35**	Female	UW 0-25%	1857	2.6% (48)	96.6% (1793)	0.9% (16)
**35**	Female	UW 25-50%	1676	19.8% (332)	80.0% (1340)	0.2% (4)
**35**	Female	UW 50-75%	1448	51.0% (739)	49.0% (709)	0.0% (0)
**35**	Female	UW 75-100%	3809	93.8% (3571)	6.2% (237)	0.0% (1)
**35**	Female	NW	21501	4.9% (1059)	85.2% (18329)	9.8% (2113)
**35**	Female	OB 0-25%	252	0.4% (1)	97.6% (246)	2.0% (5)
**35**	Female	OB 25-50%	273	0.4% (1)	63.0% (172)	36.6% (100)
**35**	Female	OB 50-75%	548	0.0% (0)	30.3% (166)	69.7% (382)
**35**	Female	OB 75-100%	3206	0.0% (0)	2.3% (73)	97.7% (3133)
**35**	Male	UW 0-25%	3475	1.7% (60)	96.9% (3366)	1.4% (49)
**35**	Male	UW 25-50%	2234	15.8% (353)	84.1% (1878)	0.1% (3)
**35**	Male	UW 50-75%	1508	45.1% (680)	54.8% (827)	0.1% (1)

**35**	Male	UW 75-100%	3040	92.6% (2816)	7.4% (224)	0.0% (0)
**35**	Male	NW	66198	1.4% (894)	84.8% (56151)	13.8% (9153)
**35**	Male	OB 0-25%	1188	0.1% (1)	93.3% (1108)	6.6% (79)
**35**	Male	OB 25-50%	2010	0.0% (0)	62.3% (1253)	37.7% (757)
**35**	Male	OB 50-75%	3754	0.0% (0)	28.8% (1083)	71.2% (2671)
**35**	Male	OB 75-100%	20211	0.0% (0)	2.6% (533)	97.4% (19678)

Values are presented as % (n) among participants with an available BMI measurement at each age. BMI categories were defined as underweight (UW) (<18.5 kg/m^2), normal weight (NW) (18.5–24.9 kg/m^2), and obesity (Ob) (≥25.0 kg/m^2). When multiple BMI records were available at the same age for the same participant, the mean BMI at that age was used for classification. Percentages may not sum to exactly 100% because of rounding.
